# A scoping review and thematic classification of patient complexity: offering a unifying framework

**DOI:** 10.15256/joc.2012.2.15

**Published:** 2012-10-10

**Authors:** Alexis K. Schaink, Kerry Kuluski, Renée F. Lyons, Martin Fortin, Alejandro R. Jadad, Ross Upshur, Walter P. Wodchis

**Affiliations:** ^1^Bridgepoint Collaboratory for Research and Innovation, Bridgepoint Health, Toronto, ON, Canada; ^2^Centre de Santé et de Services Sociaux de Chicoutimi, Chicoutimi, QC, Canada; ^3^Centre for Global eHealth Innovation, Toronto General Hospital, Toronto, ON, Canada; ^4^Department of Family and Community Medicine, University of Toronto, Toronto, ON, Canada; ^5^Institute of Health Policy Management and Evaluation, University of Toronto, Toronto, ON, Canada

**Keywords:** chronic conditions, complexity, multimorbidity, holistic health, biopsychosocial model

## Abstract

The path to improving healthcare quality for individuals with complex health conditions is complicated by a lack of common understanding of complexity. Modern medicine, together with social and environmental factors, has extended life, leading to a growing population of patients with chronic conditions. In many cases, there are social and psychological factors that impact treatment, health outcomes, and quality of life. This is the face of complexity. Care challenges, burden, and cost have positioned complexity as an important health issue. Complex chronic conditions are now being discussed by clinicians, researchers, and policy-makers around such issues as quantification, payment schemes, transitions, management models, clinical practice, and improved patient experience. We conducted a scoping review of the literature for definitions and descriptions of complexity. We provide an overview of complex chronic conditions, and what is known about complexity, and describe variations in how it is understood. We developed a Complexity Framework from these findings to guide our approach to understanding patient complexity. It is critical to use common vernacular and conceptualization of complexity to improve service and outcomes for patients with complex chronic conditions. Many questions still persist about how to develop this work with a health and social care lens; our framework offers a foundation to structure thinking about complex patients. Further insight into patient complexity can inform treatment models and goals of care, and identify required services and barriers to the management of complexity.

Journal of Comorbidity 2012;2:1–9

## Introduction

Compared with a century ago, when the most prevalent illnesses were infectious [1], 50% of the general population in developed nations now live with chronic conditions [2]. Recent acknowledgement that many patients have multiple conditions, often compounded with psychosocial challenges (i.e. complexity) [3], and that cost of care for them is very high, has positioned patient complexity in focus within healthcare policy, practice, and research.

Patients with chronic conditions cost the healthcare system billions of dollars annually [4], a finding that has been found internationally [5]. For example, medical care costs in Canada for people with chronic conditions account for 42% of total direct medical care expenditures, and 65% of indirect costs [1]. Among the indirect costs are the burden and cost to informal and family caregivers, who have contributed a value estimated at $25 billion in Canada (i.e. the cost of replacing informal caregiving with paid professionals) [6]. Another Canadian study estimated the cost to the system for those with chronic disease to be approximately double that of caring for those without a given condition [7]. Although we approached this issue with a Canadian focus, the implications are much broader. The present and projected cost estimates of providing care for these patients in the current healthcare system infrastructure have been cited as unsustainable [8].

Beyond cost is the concern about healthcare quality. In an international study of patient experience, those with multiple conditions reported poorer quality and overall dissatisfaction with their care compared with single-condition counterparts [9]. It is widely acknowledged that an acute care model is inappropriate for chronic conditions [10, 11]. As stated by Kane [12]: “Although demographic and epidemiological studies clearly show that we are firmly in the era of chronic disease, we persist in acting as though we are still treating primarily acute illnesses.” Coordination of care is cited as integral to high-quality care [13], but it is extremely challenging to organize care for people with complex chronic conditions due to the number of care providers involved [14]. Over the past 10 years, models of chronic healthcare, such as the Chronic Care Model [15] and the Expanded Chronic Care Model [16], have identified care coordination as an integral component. However, it is unclear to what extent these models have been applied to and/or successful in the management of complex patients.

The objective of this paper is to contribute to our understanding of complexity by performing a structured scoping review of descriptions and definitions of complexity in the literature. Three broad types of descriptions of complex chronic conditions were identified and are described: multimorbidity (i.e. two or more concurrent health conditions), healthcare resource utilization, and psychosocial vulnerabilities. These perspectives and the findings of the scoping review were synthesized via investigator consensus into a unifying, high-level complexity framework to aid in approaching the many questions that remain about providing optimal care for complex patients.

## The state of the literature on complexity

### Methods

A scoping review was conducted to investigate the fundamental question of what patient complexity is, and how it is currently understood in the context of providing health services and/or outcomes. Relevant databases (i.e. MEDLINE, EMBASE, Social Work Abstracts, PsycINFO, Scholars Portal, PubMed, Google Scholar) were searched in July of 2010. The following key terms were used: *comorbidity, multiple comorbid$, multiple chronic disease$, multimorbid$, polypathol$, pluripathol$, complex chronic disease$, complex chronic illness$, multiple comorbid$, complex chronic disabil$*. The above terms were searched in combination with: *care delivery, care model$, chronic care model, care manag$, case manag$, integrat$ care, coordinate$ care, care delivery model$, social care, health service$, social service$, health care*. The above search terms were selected to capture variations in spelling conventions or hyphenation (e.g. co-morbidity). Relevant references were hand searched, and principal authors contacted where appropriate. The search was limited to adults (aged 19 years or older), English articles, and research involving humans. Included articles focused on populations with two or more chronic conditions and some aspect of healthcare delivery. Articles were excluded if they focused on pediatric subjects, single-disease groups, or medical education. Abstracts were reviewed by one investigator for linkages between chronic disease, care or individual characteristics, and acknowledgement of case or care complexity. Articles that were unclear if they met the criteria for inclusion based on the abstract were reviewed in their entirety. The findings were classified into five broad health dimensions that emerged from the review. Health dimensions represented by this review include medical/physical health, mental health, demographics, social capital, and health and social experience.

## Results

The search returned 1,669 articles, 127 of which were considered relevant as assessed by the inclusion/exclusion criteria. Table 1 summarizes the breadth of the findings from the scoping review.

In exploring the literature on patient complexity, we found evidence that chronic conditions can be complex due to interconnections with many other dimensions, including, but not limited to: medical/physical challenges, such as functional impairment [10, 17–20], chronic symptoms (e.g. pain) [80], challenges in the application of clinical practice guidelines (CPGs) [10, 24–28], multimorbidity [29–42], and polypharmacy [21–23]; mental health challenges, such as depression [43, 44], psychological distress [45–47], cognitive impairment [51, 52], and substance use [53, 54]; social health issues including caregiver strain [64], poor social support [66, 67], relationship strain and lack of leisure time [63]; experiential challenges including poor quality of life [31], difficulty navigating services [78] and the need for a care manager [79], lack of access to providers [78, 79], heavy utilization of services [17, 47, 67, 69], and higher healthcare costs [1, 7, 47, 70–74]; and demographic characteristics including advanced age [55–58] and frailty [18, 22, 59], gender [40, 47], poverty [40, 47, 65], ethnic disparities [60, 61], and lower level of education [62].

In reviewing the breadth of the literature on patient complexity, it became apparent that there are three broad types of descriptions of complexity: multimorbidity, resource utilization, and psychosocial complexity. The following sections provide an overview of these perspectives.

## Complexity due to multimorbidity

Many patients experience two or more chronic conditions simultaneously (i.e. multimorbidity). Complexity may be associated with multimorbidity in many cases though it is not a necessary cause. In the literature on patient complexity, multimorbidity reigns as the most investigated description. There are several terms that have been used synonymously for this phenomenon, including: comorbidity, polypathology, pluripathology, multimorbidity, and multiple chronic conditions [81]. In 2010, an e-book across the international clinical and research community from the Observatory of Innovative Practices for Complex Chronic Diseases Management (OPIMEC) [81] was published to raise awareness about multiple chronic conditions’ prevention and management due to the growing recognition of such patients.

An international population-based survey estimated that multimorbidity affects approximately 20–30% of chronically ill patients [2]. In primary care practices, multimorbidity is more prevalent [82] than in the general population, which may be attributable to their relatively better health status, differences in access and use of services, or unmet need. Multimorbidity in primary care patients aged 65 years and older has been estimated to be as high as 97–98%, while for younger and middle-aged patients, estimates are in the order of 69% and 93–95%, respectively [29, 40, 82]. In the Netherlands, the number of patients with multimorbidity in primary care has doubled since the mid-1980s, and the proportion of patients with four or more chronic diseases has tripled [40].

Comparing estimates of multimorbidity is difficult due to variations in how the concept is defined and studied [83]. The number and type of diseases, setting, health registry characteristics, and patient demographics affect estimates of multimorbidity prevalence and incidence [84]. A recent systematic review of multimorbidity prevalence studies revealed that variation in study methodologies and findings yields dramatically different prevalence estimates, with the largest differences between studies’ estimates being up to 59% and 92% for the general population and primary care, respectively [85]. To address these issues, a more uniform methodology (e.g. operational definition, diagnoses to be included) is recommended to augment the comparability of data [85]. Yet even with comparable data, it remains unclear what proportion of patients with multimorbidity is complex in terms of the multiplicity of physical, mental health, and social issues, or in terms of complex or extensive care.

Measurements of multimorbidity tend to be simple or weighted counts of diagnoses [86]. Mercer and colleagues assert that defining complexity solely as two or more conditions underestimates the intricacy of these patients [34]. An alternative measure, the Cumulative Illness Rating Scale (CIRS), rates body systems on pathophysiologic severity as opposed to counting diagnoses. The CIRS has been adapted into an electronic assessment (eCIRS) in primary care to quantify multimorbidity [87]. De Groot and colleagues [88] critically evaluated the reliability and validity of 13 common measures of comorbidity and concluded that, in terms of capturing mortality, disease severity, type and severity of comorbidities, and functional impairment, several measures were appropriate for clinical research. As is self-evident, such measures focus predominantly on medical complexity.

Despite the occurrence of multimorbidity, healthcare and research continue to be organized mainly according to specific conditions. There are systemic contributors to this, including the training of physicians in disease-focused paradigms and the reliance on specialist practitioners; however, we chose to omit these issues in this article. Rooted in evidence, CPGs and other medical evidence predominantly address single conditions and rarely refer to or suggest a course of action for patients with multimorbidity [89–91]. Boyd and colleagues illustrated that following CPGs for an older patient with five conditions results in a complicated regimen of 12 medications at 19 doses per day, 14 non-pharmacological treatment activities, and any number of medication–diet–disease interactions [26]. Potential interactions are one way that multimorbidity may be complex. Clinicians must draw more on clinical expertise and patient preferences than CPGs to meet complex patients’ needs [28]. It is evident that caring for patients with multimorbidity is challenging for patients and practitioners, in terms of decision-making around treatment course, regimen manageability, and risk–benefit analysis.

## Complexity in terms of healthcare utilization

A system-oriented description of complexity in chronic conditions is from the perspective of healthcare resource consumption. A study of high users of healthcare by Reid and colleagues found that the 5% who use the most resources, including primary care, specialist encounters, and inpatient days, are characterized by multiple, complex health issues [47]. The Kaiser Permanente Chronic Conditions Management pyramid is one model that recognizes this heterogeneity in the chronically ill population; differentiating non-complex from complex patients (i.e. multiple chronic conditions, risk of decline, fluctuating or poorly managed conditions) [92]. The Kaiser model indicates that patients at the top of the pyramid are the most complex, as defined by historical healthcare use and disease registries, and thus targets intensive case management to these complex patients [92]. There are a number of adaptations of the Kaiser model, including the UK’s National Health Services Health and Social Care Long Term Conditions Model. In the Kaiser model, aside from health history, little is known about the characteristics of these patients that make them complex.

Resource use is commonly estimated via case–mix indices. These indicators are the product of an algorithm that stratifies patients into relatively homogeneous clusters. An example is the Johns Hopkins Adjusted Clinical Groups (ACG) methodology, which quantifies morbidity using diagnostic codes and groups patients by age, gender, and expected consumption of healthcare resources and short-term clinical outcomes (see [93]).

Complex chronic conditions typically necessitate ongoing clinical management, are associated with poor health outcomes, greater utilization of healthcare services, and higher cost [74]. However, the question must be raised if high healthcare resource use is indicative of a complex patient. The potential circularity of resource utilization equating to complexity, and care need, has been summed up by Wade: “If complexity is defined operationally as equivalent to needing or using more resources, then obviously more complex cases will need or use more resources!”[3]. Alternatively, patient need may be underestimated where services or professionals (e.g. home care, psychologists) are not available or service provision is inadequately documented. Thus, caution must be exerted when relying solely on resource utilization to indicate complexity.

## Complexity due to psychosocial factors

It is well established that chronic illness is not just about the disease, but intersects with the broader socioeconomic context. An estimated 75% of the factors that influence health lie outside of the healthcare system [94]; for instance, poverty and social exclusion are key chronic condition risk factors. Health inequities are closely tied to health status and wellbeing [95], and chronic conditions can force individuals into poverty due to the extensive cost of treatment [96]. A study of primary care physician’s ratings of complexity for patients in their practices revealed that over 25% of patients were considered complex by their physicians, with socioeconomic and behavioral factors driving complexity ratings more than medical diagnoses [97]. It is increasingly evident that any conceptualization of complexity is incomplete without consideration of social and environmental factors.

Psychosocial complexity tends to refer to one or some combination of social isolation, psychiatric illness, socio-demographic vulnerability, or other social and/or psychological difficulties [98]. This may include persistent distress or fear that is not adequately addressed and complicates medical management [98]. Nardi and colleagues [22] analyzed complex internal medicine patients, and suggest two key variables that load onto the construct of complexity: disability in physical function and frailty (i.e. a condition with a high risk of negative outcome and worsening quality of life commonly associated with disability and socioeconomic issues).

In response to psychosocial complexity, Safford and colleagues [99] developed a model including population-level determinants of health (i.e. socioeconomics, culture, behavior, and environment). Their Vector Model of Complexity depicts each determinant as a vector influencing the direction (i.e. increase or decrease) and magnitude of a patient’s complexity. The interconnectedness of the determinants is outlined, whereby a disturbance of any one affects the entire balance [99]. The Vector Model may account for some of the key challenges practitioners face with complex patients, such as why the same treatment plan is poorly adhered to or differentially effective for different patients (i.e. the treatment is not sufficiently individualized with a shared vision of realistic outcomes). The need for CPGs to include guidance on tailoring treatment plans is emphasized [99], an idea central to collaborative care (e.g. [63]). A shortcoming of this potentially useful model is the application of mathematical vectors to mathematically devoid determinants of health, thus, yielding the angles between vectors meaningless for quantification of complexity [100].

In the same vein, there are few clinical measures that tap into biopsychosocial definitions of complexity. Of interest, the INTERMED Complexity Assessment Grid systematizes a biopsychosocial approach to ascertain case complexity [101]. The INTERMED was developed to operationalize the biopsychosocial model and identify characteristics that pose obstacles to care, using a combination of psychometric and clinimetric theories to maintain the reliability and validity, and relevance and brevity, respectively, of each approach [101]. The tool makes it possible to identify the need for multidisciplinary intervention for patients who may respond poorly to conventional biomedical interventions [101], and has been successful in identifying clusters of complex patients [102] and improving health outcomes for patients (e.g. [103, 104, 105, 106, 107]). Although the tool has been applied to several patient populations in a variety of settings, including the adoption of an adapted version by the Case Management Society of America, it has not yet seen system-level uptake.

## Synthesis into a Complexity Framework

**Figure 1:**
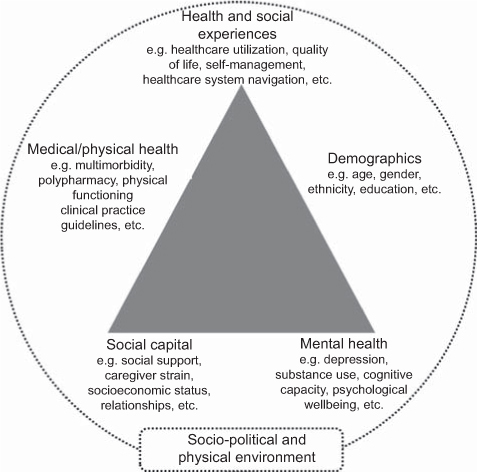
The Complexity Framework showing five health dimensions.

Although they tend to be reported in separate bodies of literature, it appears that multimorbidity, resource use, and psychosocial vulnerability are closely intertwined in understanding complex patients [32]. There are many possible permutations of these factors, thus, a high degree of uniqueness among complex patients; a challenge to the healthcare system that draws largely on the application of rules to homogenous patient groups. As a synthesis to our review process and to consolidate thinking, we developed a high-level framework to guide our own research development and approach to complexity (Figure 1). Existing models were drawn upon for their contributions to understanding complex patients.

The scoping review informed the development of this framework via thematic analysis of the literature and investigator consensus reached through discussion. The framework is centered on the top of the Kaiser Permanente Chronic Conditions Management diagram, with the triangle in our framework representing the 1–5% most complex chronically ill patients identified in the Kaiser model. The Kaiser model identifies patients at the top of the pyramid as the most resource intensive, yet there is little understanding what personal or experiential characteristics make them a high-risk population. In an effort to fill this gap in understanding, the framework broadly synthesizes the scoping review findings around the question: *What needs to be considered to gain an understanding of patients with complex chronic conditions?* Thus, the framework includes the five health dimensions detailed in Table 1, and examples of the types of granular elements are provided for illustrative purposes.

Our Complexity Framework builds on past descriptions of complexity, synthesizing a number of perspectives, literature, and adding a unifying, high-level approach. Considering the framework dimensions concurrently offers a systematic and holistic perspective with which to understand patient challenges, goals, and care need in research that can ultimately inform policies to support clinicians in caring for complex patients. Our search results are limited in scope by the terms we used. Broadening the search to include other terms, such as *disability* and *frailty*, could provide further insight into these factors and their relation to patient complexity. Further research is required to validate the framework, and we are beginning by using it to guide our program of research to test the hypothesis that these dimensions are salient for inpatients receiving post-acute complex care and rehabilitation, and for outpatients at other points in the continuum of care in a Canadian context. In addition to our conceptualization of patient complexity, there are systemic and structural issues that add another layer of complexity that may manifest as care-regimen complexity, healthcare system navigation challenges, or complex networks of healthcare providers. The framework should stimulate further thought, discussion, and empirical study of complexity and bring together experts and decision makers at the organizational and regulatory levels to proactively address complexity in healthcare settings.

In this paper, we describe three dominant general understandings of complexity, and synthesize the literature in a complexity conceptual framework. Key questions remain around how a holistic and systematic understanding of complexity could inform the development of treatment models and required skill mix for clinicians who provide care to patients with complex chronic conditions. It also remains unknown if such an approach can provide a better understanding of patient goals of care and identify the resources and programs required. Answering such questions will help to identify barriers to the effective management of complexity.

## Figures and Tables

**Table 1:** Examples of elements of complexity classified by health dimensions identified via scoping review.

Dimension	Example	Sources
Medical/physical health	Loss of physical functioning Polypharmacy Limited application of clinical practice guidelines Multimorbidity	[10, 17–20] [21–23] [10, 24–28] [29–42]
Mental health	Psychological distress Psychiatric illness (e.g. depression) Cognitive impairment Addictions/substance use	[43–47] [41, 43, 48–50] [51, 52] [53, 54]
Demographics	Older age Frailty Female gender Ethnic disparities Lower education	[55–58] [22, 59] [40, 47] [60, 61] [62]
Social capital	Negatively affected relationships Caregiver strain and burnout Low socio-economic status and poverty Poor social support	[63] [64] [40, 47, 65] [66, 67]
Health and social experiences	Heavy utilization of healthcare resources Costly careSelf-management challenges Poor quality of life Difficulty with healthcare system navigation	[17, 47, 68, 69] [1, 7, 47, 71–74] [23, 69, 75–77] [31] [78, 79]
